# Potential of Melatonin as Adjuvant Therapy of Oral Cancer in the Era of Epigenomics

**DOI:** 10.3390/cancers11111712

**Published:** 2019-11-02

**Authors:** Ana Capote-Moreno, Eva Ramos, Javier Egea, Francisco López-Muñoz, Emilio Gil-Martín, Alejandro Romero

**Affiliations:** 1 Department of Oral and Maxillofacial Surgery, University Hospital La Princesa, Autonomous University of Madrid, 28006 Madrid, Spain; anacapotemoreno@gmail.com; 2 Department of Pharmacology and Toxicology, Faculty of Veterinary Medicine, Complutense University of Madrid, 28040 Madrid, Spain; eva.ramos@ucm.es; 3 Health Research Institute, Clinical Pharmacology Service, University Hospital La Princesa, Autonomous University of Madrid, 28006 Madrid, Spain; javier.egea@inv.uam.es; 4 Institute Teófilo Hernando for Drug I+D, School of Medicine, Autonomous University of Madrid, 28029 Madrid, Spain; 5 Faculty of Health Sciences, University Camilo José Cela, 28692 Madrid, Spain; flopez@ucjc.edu; 6 Neuropsychopharmacology Unit, Hospital 12 de Octubre Research Institute (i+12), 28041 Madrid, Spain; 7 Portucalense Institute of Neuropsychology and Cognitive and Behavioural Neurosciences (INPP), Portucalense University, R. Dr. António Bernardino de Almeida 541, 4200-072 Porto, Portugal; 8 Thematic Network for Cooperative Health Research (RETICS), Addictive Disorders Network, Health Institute Carlos III, MICINN and FEDER, 28029 Madrid, Spain; 9 Nutrition and Food Science Group, Department of Biochemistry, Genetics and Immunology, Faculty of Biology, University of Vigo, 36310 Vigo, Spain

**Keywords:** oral cancer, melatonin, adjuvant treatment, melatonin receptors, epigenetics, methylation, chromatin modification, miRNAs

## Abstract

The wide variety of epigenetic controls available is rapidly expanding the knowledge of molecular biology even overflowing it. At the same time, it can illuminate unsuspected ways of understanding the etiology of cancer. New emerging therapeutic horizons, then, promise to overcome the current antitumor strategies need. The translational utility of this complexity is particularly welcome in oral cancer (OC), in which natural history is alarmingly disappointing due to the invasive and mutilating surgery, the high relapsing rate, the poor quality of life and the reduced survival after diagnosis. Melatonin activates protective receptor-dependent and receptor-independent processes that prevent tissue cancerisation and inhibit progressive tumor malignancy and metastasis. Related evidence has shown that melatonin pleiotropy encompasses gene expression regulation through all the three best-characterized epigenetic mechanisms: DNA methylation, chromatin modification, and non-coding RNA. OC has received less attention than other cancers despite prognosis is usually negative and there are no significant therapy improvements recorded in the past decade. However, a large research effort is being carried out to elucidate how melatonin´s machinery can prevent epigenetic insults that lead to cancer. In the light of recent findings, a comprehensive examination of biochemistry through which melatonin may reverse epigenetic aberrations in OC is an extraordinary opportunity to take a step forward in the clinical management of patients.

## 1. Introduction

The oral cavity is the anatomical place where approximately 50% of head and neck cancers (HNC) appear. Oral cancer (OC) can originate in the epithelium covering any anatomical structure of the oropharyngeal and nasal cavities. The oral squamous cell carcinomas (OSCC) and oropharynx mucosa, also known as epidermoid oral carcinoma, are the most frequent malignant diseases of the upper aerodigestive tract, representing 90–95% of new diagnoses of oral malignancies [1].

The poor prognosis and high mortality that still characterize OC, combined with the lack of better future perspectives, is a challenging panorama. The unreliability of screening tests or sensitive biomarkers for a precise diagnosis, together with the unavailability of effective therapeutic tools hampering malignant progression and drug resistance, add concern to the current situation. In this regard, the clinical outcome of OC patients is unpredictable and life threatening, leading to unsatisfactory 5-year survival rates (~50%), which has remained unchanged in recent decades [2,3]. This unfavorable clinical prognosis results from the high tendency of oral tumors to proliferate rapidly, doubling in size every ~6–7 days [4], the development of locoregional recurrence, second primary tumors and radiochemotherapy resistance. As a result, the tumor cells spread out to secondary tissues (mainly the lungs, liver and bones) [3], diminishing the survival of OC patients [3]. 

Up to an 80% of OC patients display unpredictable and erratic outcomes in response to radiochemotherapy, which is related to genetic variability. The heterogeneous spectrum of interindividual epigenetic changes [5] makes the assessment and therapeutic management of this disease difficult. Therefore, the knowledge of the epigenetic pathways behind oral malignancy would offer a great opportunity for personalized treatments and patient follow-up [6]. In this regard, the large amounts of data obtained from current molecular technologies facilitates the design of an OC meta-signature based on its epigenetic features [7]. It is expected that in this way a new generation of biomarkers will allow improved early and highly predictive diagnoses, enabling tailored therapies directed to the epigenetic idiosyncrasy of each tumor [8]. Consequently, the development of robust multiplexing predictors may potentiate the clinical outcome and quality of life of OC patients [9]. The understanding of the epigenetic map of OC is therefore a necessary step to make the clinical practice more effective, allowing it to evolve from histopathology to a molecular-based era [10].

In this complex scenario, we will focus special attention on the role of melatonin as an adjuvant treatment in OC therapy. Although under physiological conditions melatonin is mainly released from the pineal gland, other significant extrapineal sources of melatonin such as the enteroendocrine cells of the gastrointestinal tract, including the oral cavity and the salivary glands should be considered too [11]. Albumin-free blood melatonin passively diffuses via saliva into the mouth, where it reaches amounts 70% lower than in circulation [12]. In vertebrates salivary melatonin levels oscillate according to the rhythmicity observed in the serum [13], from a diurnal 1–5 pg/mL to 50 pg/mL at night [12]. Saliva is acquiring a great deal of attention as a preferential milieu to search for OC biomarkers [14,15]. Simultaneously, it is an excellent route for the direct administration of melatonin, alone or combined with standard therapeutic strategies. In this regard, melatonin enhances radiochemotherapy cytotoxicity by stimulating intracellular generation and accumulation of reactive oxygen species (ROS), apoptosis and autophagy [16,17], while in parallel melatonin antagonizes several undesired side effects on the oral mucosa [18,19]. It is of great translational importance to undertake blinded, independent and randomized controlled trials that corroborate the apparent protective effects on the oral cavity of local and systemic melatonin. The lack of toxicity and side effects reported by the different studies conducted in vivo, as well as the amelioration of life quality and survival of OC patients urge those melatonin studies. Last but not least, melatonergic treatments are inexpensive when compared to the standard radiochemotherapy currently in use, and this reinforces the necessity to deeply explore the clinical utility of this indoleamine.

## 2. Epidemiology of Oral Cancer

OC is a growing public health concern due to its worldwide over-increasing incidence in developed and especially in developing countries (3/4 of cases). Indeed, every year some 550,000 new cases are diagnosed in the world [20], which make it the 6th most frequent carcinoma [1,21] accounting for ~2% of all new diagnoses worldwide [4]. The incidence and mortality are particularly alarming in certain transitioning regions, such as South-Central Asia, where the neoplasia is the most common malignancy and the 2nd cause of cancer death among men [22,23]. In Europe HNC represents about 4% of all arising cancers, with a census of 140,000 new cases and 63,500 deaths in 2012 [21]. In the United States statistics from 2014 reported 55,070 new cases of oral cavity, pharyngeal and laryngeal cancers and 12,000 deaths [22], with similar estimates (53,000 new cases and 10,860 deaths) for this year according to The American Cancer Society [23].

It is especially worrisome that OC incidence depends highly (up to 75%) on lifestyle habits such as alcohol and tobacco consumption and chewing betel nut [24], as well as a low socioeconomic status leading to poor nutritional health, the presence of premalignant lesions, or human papillomavirus (HPV) infection [25]. The most carcinogenic serotypes, HPV-16 and HPV-18, have been well documented as prognostic factors for oropharyngeal carcinomas [26,27]. However, HPV positive oropharyngeal tumors show a different behavior and have therefore been included as an independent HNC group by the last 8th Tumor-node-metastasis (TNM) classification [28]. Nevertheless, the carcinogenic influence of HPV in other oral locations such as the tongue or floor of the mouth remains to be fully elucidated [27].

Although the onset of disease is more frequent between the 4th to 6th decades of life, recent studies have reported contradictory census data. While some surveys suggest a fall in the age-standardized incidence of OSCC [26], others have reported a higher prevalence in patients older than 65 [29]. Likewise, despite the fact men have traditionally been associated with a high incidence of OSCC, recent studies have demonstrated an increase in the number of cases in women [29,30]. This issue may be related to the growing popularization of smoking and alcohol consumption among women that has occurred over the last two decades.

Despite intense efforts to improve diagnostic techniques and therapy, the 5-year survival remains at around 50% [31]. This poor prognosis is related to the classical risk factors, as well as the tumor size, neck lymph node metastases, surgical margins, or a deep tumor invasion [29,32]. Like HPV positivity, the depth of tumor invasion has led to changes in the last TNM staging of HNC [28]. Neck status has been widely recognized as one of the most determinant variables affecting OSCC evolution [33,34]. Neck positive nodes at primary treatment drastically drop survival rates to below 50%, thus current protocols are focused on early lymph node detection and treatment. Similarly, the high locoregional recurrence worsens the prognosis and decreases the overall survival of OSCC patients. Undoubtedly, massive screening programs, an adequate prophylaxis of oral cavity as well as the cessation of substance abuse, are unavoidable requirements for ameliorating the alarming epidemiology of OC. However, health education or efficient visual screening [35] will not be enough to address the clinical challenge posed by an initially insidious cancer, which symptomatology debuts in a malignant and usually aggressive phase. For these reasons, to overcome the unacceptable morbimortality rates, understanding epigenetic remodeling and mechanistic abnormalities and finding effective serological or tissue biomarkers are essential requirements.

## 3. The Role of Melatonin in the Oral Cavity: Functionality and Alterations in Oral Cancer

The beneficial role of melatonin for the management of many cancers has been widely proposed [36,37,38]. However, the potential benefits of melatonin in the treatment of oral carcinomas are less well known and it is therefore, imperative to concentrate research in this area.

**Figure 1:**
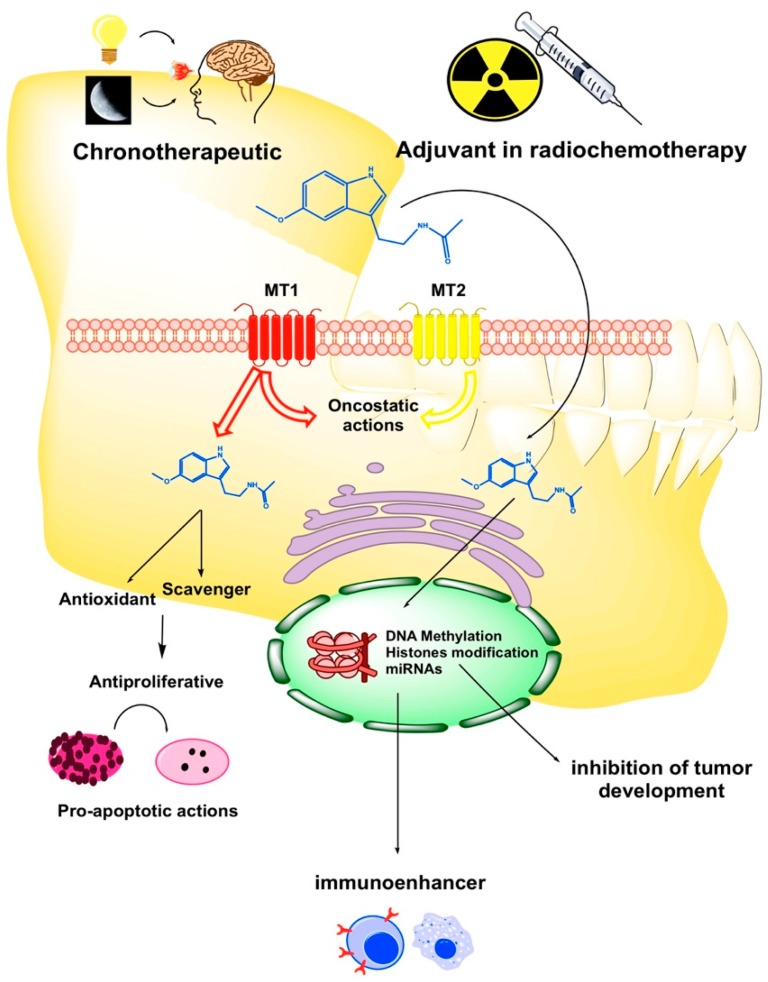
Schematic diagram of the critical points where melatonin may exert its protective and therapeutic effects on oral cancer (OC). MT1: melatonin receptor type 1; MT2: melatonin receptor type 2.

Oxidative stress (OS) and ROS generation are two pivotal factors involved in oral cancerisation through: (i) The damage of the DNA and proteins of normal keratinocytes; (ii) the oncogenic mutations and malignant transformation of oropharyngeal mucosa and (iii) the enhancement of progression and invasiveness of OC cells. In this regard, melatonin displays oncoprotective and oncostatic activities due to its antioxidant properties as a powerful scavenger of ROS [39] (Figure 1). Under hypoxic conditions tumor cells express the hypoxia inductive factor 1-alpha (HIF-1α), which induces the overexpression of pro-angiogenic vascular endothelial growth factor (VEGF) involved in tumor progression and metastases [40]. In this context, melatonin strongly inhibits VEGF and other stress factors such as epidermal growth factor (EGF) or insulin growth factor-1 (IGF-1) [40]. Thus, melatonin interferes with tumoral cell proliferation and tumor growth and acts as a pro-apoptotic agent in tumoral keratinocytes [39,41]. Proof of the anti-tumor activity of melatonin has been recently reported citing the association of the melatonin synthesis/metabolism index in the tumor microenvironment, with reduced somatic mutations and neoantigen expression in head and neck squamous carcinomas [42]. 

The innate and adaptative immune cells are efficient cytotoxic anti-tumor defenses. However, eventually tumor cells evade immune surveillance by controlling cellular apoptosis and initiate the tumor cycle that leads to the development of cancer and tumor progression. In this regard, it has been described a cell-mediated immune depression in HNC, even after surgical resection [43]. Melatonin is a potent immunoenhancer that increases the activity of T and B lymphocytes, monocytes and natural killer (NK) cells and stimulates the secretion of cytokines (interleukin-2 (IL-2), interleukin-6 (IL-6), interleukin-12 (IL-12) and tumor necrosis factor-α (TNF-α)) (Figure 1), thereby leading to oncostasis and the inhibition of tumor spreading [24].

## 4. Responses Mediated by Melatonin Receptors in Oral Cancer

According to the classification of the International Union of Pharmacology, two high-affinity G protein-coupled receptors, named MT1 and MT2, represent the main melatonin-binding sites in mammals [44]. The pleiotropic functionality of melatonin in OC includes the activation of crucial signaling cascades, harmonizing cell homeostasis and tissue microenvironment, and the inhibition of tumor progression. MT1 and MT2 are extensively distributed in tissues and organs such as the brain, retina, gastrointestinal tract, blood vessels, ovarian, testes, kidney and adipose tissue [45]. Particularly in the oral cavity, melatonin receptors are present in the cells of salivary gland ducts and acini, oral epithelium and the osteoblasts of maxillary alveolar bone, among other oral localizations [45,46]. 

There are compelling findings that indicate that receptor signaling is pivotal in modulating gene expression, repressing OC growth and invasion [47,48] and reducing the susceptibility to environmental carcinogens [49]. Thus melatonin resembles more the functional profile of a cell protector than a classical hormone [41]. MT1 is expressed and active in healthy mucosal cells from the oral cavity, but eventually functionally cancelled in OC cell lines [41,47,50,51], highlighting the oncostatic potential of melatonin. Furthermore, recently evidence has shown that melatonin-induced apoptosis enhances the antitumor effect in tongue squamous cell carcinoma (TSCC) through the inhibition of melatonin receptor type 2 (MT2) -Transcription Factor E3 (TFE3)-dependent autophagy [52]. Indeed, the radiation of rat tongue reduced MT1 and MT2 expression, while the administration of melatonin-based gel reverted MT1/MT2 depletion [53]. In this respect, although melatonin receptors ranged from pico-nanomolar affinity, high doses of the indoleamine have been therapeutic, deprived of toxic effects and capable of increasing the expression of MT1/MT2 receptors. Related to the relevance of MT1 and MT2 activation, further research is needed to elucidate the complexity of receptor-mediated actions of melatonin and thereby outline the therapeutic potential of melatonin signaling in OC cells. 

## 5. Melatonin in Combination with Conventional Treatment for Oral Cancer and Safety Profile

One of the main concerns regarding the research and therapy in the field of oncology is the toxicological evaluation and risk assessment of new treatments directed against tumors. In the case of melatonin, it has recently been reported that even at high doses, and aside from some rare exceptions, both physiological and pharmacological concentrations have no significant toxicity [54]. 

The multiple modes of action displayed by melatonin in tumor oral cells, such as pro-apoptotic, antiproliferative and antimetastatic (Figure 1), make melatonin adjuvant therapy combined with conventional radiochemotherapy an excellent option for successful cancer treatment. There are not enough short- or long-term translational studies investigating the therapeutic potential of melatonin in clinical practice. It is known that this indoleamine is beneficial for oral oncostasis and tumor inhibition and that melatonin disruption is a predisposing factor in OC. The paucity of studies addressing the administration of melatonin in combination with other drugs for the management of OC is summarized in Table 1. Moreover, oral precancerous conditions such as leukoplakia and lichen planus also respond positively to melatonin administration, because OS is central to their etiopathogenesis [55]. There is a growing evidence of the potential of this natural occurring indoleamine in OC chemoprevention, as an adjuvant to the cytotoxicity of standard therapies and improving the welfare of the patients. Thus, melatonin treatment enhances the efficacy and reduces the toxicity of chemotherapy in patients with lung, breast, gastrointestinal tract, as well as head and neck cancers [56]. Indeed, an adjuvant therapy with high concentrations of melatonin was able to potentiate in vitro and in vivo effects of 5-fluorouracil (5-FU) [57], cisplatin and radiation-induced cytotoxicity in head and neck squamous cell carcinoma [17].

The low bioavailability of oral melatonin has prompted the development of different galenic formulations, such as topical preparations (Table 2). Thus, the application of a 3% melatonin gel prevented oral mucositis in rats subjected to radiation [53]. In the same way, a recent randomized, double blind clinical study of combined topical and oral melatonin as an adjuvant therapy in patients with HNC, has shown to reduce mucositis [58]. Nonetheless, additional clinical trials with different dose regimens and administration schedules should be tested to determine the optimization and standardization of local applications.

## 6. Epigenetic Regulation of Melatonin in Oral Cancer

There is not a unified etiological model accounting for all diagnosed OC cases [59]. In contrast, molecular defects on oncogenes and tumor suppressors that lead to genetic reprogramming of OC had been recently extended with the epigenetic aberrations in key homeostatic genes. Specifically, a mechanistic triad integrated by chemical modifications of DNA and associated proteins, in addition to non-coding RNAs inscribes heritable changes in the genome without affecting the coding sequence [60]. Thereby epigenetic marks modulate genome transcription and control some relevant cancer hallmarks. Melatonin is also in focus as a likely relevant mediator of epigenetic effects on OC cells (Table 3).

### 6.1. Epigenetic Methylation of DNA

Even though OC epigenomics is still in its infancy, the carcinogenic importance of impairing certain tumor suppressors by an extensive promoter CpG methylation has increased incessantly in recent years. Cellular and molecular evidence from OSCC have proven that transcriptional silencing is as relevant in halting gene expression as the classical point mutations or the loss of heterozygosity [59,61,62]. In this regard, putative suppressors are consistently active in healthy oral mucosa, while they are functionally cancelled in OSCC lines or malignant tissue either by double deletion or epigenetic silencing by abnormal DNA hypermethylation. The confidence in the clinical utility as a tumor biomarker of the gene-methylation profiling (hypermethylome) has spurred research and made it possible to identify additional accredited or presumed tumor suppressors involved in cell homeostasis and gene methylation maintenance. In the search for switched-off suppressors, the detailed examination of OSCC lines for bi-allelic deleted regions found a common ~1 Mb genetic loss at 4q35 where the melatonin receptor 1A gene is mapped (Table 3), thus suggesting MT1 as a new candidate for tumor suppressor in OSCC. Accordingly, the mRNA for MT1 was consistently lost or reduced in deleted lines, while in non-homozygously deleted tumor lines the MT1 promoter hypermethylation was inversely correlated with MT1 expression [50]. Moreover, the tissue from primary OSCC tumors showed that the methylated promoter MT1 and the lack of MT1 were significantly associated with poor prognosis. All the above suggests that i) OC methylotype shows specific altered DNA methylation patterns in the course of tumor initiation and progression [59]; ii) the promoter hypermethylation is a leading cause of tumor suppressors’ inhibition in OC pathogenesis [63]; and iii) the loss of function of the high affinity MT1 may be pivotal in the etiopathogenesis of OC. However, research has also reported that the global OC genome is hypomethylated and increasingly hypomethylated in univariate association with tobacco/alcohol history and the malignant stage of the tumor [64,65]. Nevertheless, the individual DNA methylotype maintains translational interest for molecular staging, prognosis and customized management of OC patients [66,67,68,69]. In support of this are the recent results obtained by connecting the alteration of the DNA methylotype of HNC patients with gene expression profiling to screen their biomarker potency [70,71]. Similarly, LINE-1 hypomethylation in peripheral blood mononuclear cells (PBMCs) when compared to control PBMCs has recently proved to be an excellent OC diagnostic biomarker of maximum sensitivity and specificity [72].

Light pollution at night (light-at-night or LAN) has recently provided evidence for the potential to facilitate cancer. Prominently, LAN alters the quality of sleep and presumably activates some putative mechanisms underlying malignant transformation, including the modification of global DNA methylation through pineal melatonin [73]. In human oral mucosa is critical the melatonin-dependent and one-day rhythmic expression of the clock gene and tumor-suppressor *PER2*, as well as its rapid adaptation to wavelength changes [74]. Therefore, as it is known that circadian alterations promote some prevalent neoplasias [75], that chronodisruption induces epigenetic abnormalities [76] and that melatonin counteracts the carcinogenic effects of circadian impairments [77,78,79], the time has now come to undertake a rigorous study of the circadian machinery and melatonin chronomodulation in OC (Figure 1). The mechanism of how melatonin contributes to the epigenetic remodelling of methylome in OC cells requires a deeper research. In this regard, it is admissible that melatonin participates in the reversal of epigenetic impairments that deteriorate physiological transcription during oral malignisation and OC development.

Due to its magnitude, dynamic nature and variability of regulatory mechanisms DNA methylation adds much complexity to the molecular etiology of OC. DNA methylotype is extensive, because it affects a large number of genes (around 1300 according to estimates of a recent survey) [80], dynamic, because the panel of methylated genes in primary oral tumors does not match that found in the metastases of the same patients [78] and variable because significant differences are detected in the interindividual DNA methylation phenotype of OC patients [59]. Particularly in OC, the dynamic and variable character of gene methylation makes it necessary to screen the methylome from tumor and paired-healthy tissue specimens, because certain habits such as smoking have been shown to increase DNA methylation of oral mucosa in non-negligible percentages [81]. Moreover, the standardization of methylotype from tumor and matched-healthy tissue genotyping can help to prevent the other debated inconsistencies, such as the upregulated DNA methylation found in premalignant oral lesions [82] and transitional tissue [83], pointing out that epigenetic disturbances presumably occur in the early phases of OC carcinogenesis.

### 6.2. Epigenetic Modification of Chromatin Structure

The catalogue of epigenetic mechanisms set in motion by melatonin is not limited to promoter hypermethylation; chemical modifications of chromatin-associated proteins respond to the same mode of controlling gene expression with no alteration in the base-pair sequence of DNA. This is the case of lysine-specific histone acetylation/deacetylation within the N-terminus protruding from the nucleosome core, that switches chromatin’s structure between the permissive (acetylated), and the highly condensed repressive (deacetylated) transcriptional states [84]. In this regard, hypoacetylation of acetyl-histone H3 at Lys9 has been associated with oral cancerisation and the poor prognosis of OC patients [85]. For the same reason, an inadequate activation of histone lysine-specific demethylase (LSD1) enhanced cancer development and worsened the prognosis of tongue squamous cell carcinoma [86]. Recently, LSD1 has been found upregulated in both clinical OSCC samples and null mice xenografted with implants from lymphoid metastatic OSSC patients [87] and LSD1 overexpression correlated with negative cancer evolution [86,87]. In congruence with this evidence, the inhibition or repression of tumor promoting LSD1 brings about histone methylation and can lead to cancer arrest. Of note, melatonin has demonstrated in a mouse preclinical model of patient-derived OSCC xenografts that it has the potential to arrest cell cycle (at G0/G1 phase) and inhibit tumor proliferation by inducing histone H3 acetylation at Lys 4 and 9 through LSD1 suppression in both tongue and gingival cancer cells [87] (Table 3). It is highly remarkable that melatonin showed this oncostatic potential on patient-derived xenografts to a similar extent as 5-FU, but is devoid of any undesirable cardiotoxic side effects. Likewise, the inhibition of histone deacetylase induced by melatonin has revealed to be efficient in reducing the radiotherapy-induced mucositis in radiated hamsters [88], as well as in patients affected by OC and other head and neck carcinomas receiving concurrent chemoradiation [58,89], which reinforces the pleiotropic benefits of melatonin administration. 

In HSC-3 and OECM-1 OC cells treated with melatonin (0–1 mM) for 24 h suppressed the expression and activation of proteolytic matrix metalloproteinase 9 (MMP-9) throughout the downregulation of extracellular signal-regulated 1 and 2 (ERK1/2) mitogen-activated protein (MAP) kinases kinases, which induced the reduction of transcriptional coactivators CREBBP/EP300 and thereby the depletion of active pool of MMP-9 by attenuating histone acetylation [90] (Table 3). This process abrogates the active transcriptional complexes on MMP-9 promoter-deprived OC cells of this crucial extracellular matrix-degrading enzyme and consequently attenuates the propagative and metastatic capacities of OC tumors. Consistent with the foregoing, MMP-9 and CREBBP/EP300 are significantly overexpressed in the tumor tissue of paired tumor/non-tumor OC surgical specimens [90]. In a similar way, millimolar melatonin reduced the migration and metastatic invasion of nasopharyngeal carcinoma cells through the suppression of MMP-9 expression by inhibiting the binding of the transcription factor, specificity protein-1 (SP-1) to DNA at the MMP-9 promoter [91] (Table 3). Thus, another epigenetic action of melatonin in OC is the protection of the internal environment of oral tissues, which enzymatic destruction precedes the departure of metastatic cells.

### 6.3. Non-Coding micro-RNAs 

The triggering of OC pathogenesis may include the dysregulation of part of the ~2500 human [15] non-coding short (~22 nt) micro-RNAs (miRNAs), which bind complimentary target mRNAs and initiate their degradation or repression via RNA interference. Directly or indirectly, it is estimated that each miRNA can modulate thousands of genes through negative translational regulation [92], so that expressed miRNAs are indicative of the functional profile of the cell. It is thus plausible that miRNAs can modulate crucial pro- and anticarcinogenic genes with oncogenic or suppressive roles in early OC [93]. Given that precursor and mature miRNA expression is tumor and tissue specific, the numerous studies that have been undertaken during the last few years have revealed multiple altered miRNAs in oral diseases, premalignant lesions and OSCC [15,94,95,96]. Deregulated miRNAs in OC include several dozens of up-regulated oncogenic miRNAs (OncomiRs) and depleted miRNAs oncosuppressors [95], eventually within wide dynamic ranges of variation. More importantly, the unveiled association with development, clinicopathological features, progression and/or therapy resistance of oral tumors has led to the proposal that some miRNAs are putative predictors of early carcinogenesis and outcome of OC patients [95,97]. 

The impact of melatonin on miRNA expression in paired healthy/tumor OC tissue has been scarcely addressed (Table 3). Attention has been recently focused on oncogenic miRNA-24, up-regulated in OSCC cells with respect to normal keratinocytes, in tumor tissues of the oral cavity relative to healthy ones, as well as in the plasma of OSCC patients [98]. On the other hand, in MCF-7 cells, 22 miRNAs differentially modulated by physiological nanomolar melatonin [99], including down-regulated miRNA-24 [100], may be held partially responsible for the antitumor effects of the indoleamine [101]. In this regard, micromolar melatonin induced the repression of miRNA-24 and almost reversed the activation of a four-gene panel shared by melatonin/miRNA-24 pathways [100], which aided the inhibition of tumor cell proliferation and the migration of colon, breast and head/neck tumor cells. It is therefore of great interest to investigate the effect of melatonin on miRNA-24 in the tumoral phenotype of OC cells.

Other miRNAs targeted by melatonin in OC have recently been identified in the baseline content of exosomes from SCC9, SCC25 and CAL27 lines [102]. As previously performed using tumor lines profiled for expressed miRNAs [103], this pioneering study described the exosomal signature of miRNA secreted by different OC cells and their different modulation by micromolar melatonin for 72 h. Specifically, the expression of miR-155 dropped while the presence of miR-21 raised in all the exosome fractions analyzed. miR-21 is a recognized OncomiR in OC [104,105,106,107], which is highly overexpressed in tumor lines [108] and tissue samples [109] and associated with a negative prognosis in OC patients [110]. In this context, it has not only been reported that miR-21 is overexpressed in direct exfoliation cells of tumor foci [109] and in tissue specimens and oral swirls [111] of both OC patients and the controls, but also, this up-regulation has been directly associated with the cancerisation of oral tissues [109]. The latest report of up-regulated miR-21 was in the exosomal content of OC lines exposed to melatonin is surprising [102]. Certainly, gene and miRNA expression are under the control of multiple mediators and melatonin could be only one of the actors modulating the expression of miR-21. Nevertheless, the apparent influence of melatonin on miR-21 expression in the exosomes emitted by tumor cells of oral cavity highlights the active role that melatonin may play in the control of oral carcinogenesis.

The panel of genes epigenetically targeted by melatonin have a high translational value for OC therapy although they remain virtually unexplored. For example, their biological potential as early reporters of OC pathogenicity of paracrine miRNAs originating from damaged cells, or from active exosomal secretion is extraordinary, but unfortunately, the influence of pleiotropic melatonin on their functional biology has not been studied in depth. Nonetheless, the incentive of easily assessable biomarkers on read-out platforms catalyses the ongoing efforts to setting miRNAs and remaining epigenetic marks on specific palettes, for the screening of diagnosis, prognosis and personalized therapy of OC.

## 7. Conclusions

Oral cancer agglutinates the neoplasms of oral cavity, which are worryingly prevalent worldwide, and have an important environmental etiology. Of particular concern is the silent development of incipient tumors, their rapid progressivity and resistance to standard radiochemotherapy. The lack of morphological and molecular methods for the detection of preneoplastic lesions, or early tumors, exacerbates the clinical outcome of the disease and adds urgency to the development of new therapeutic approaches. Melatonin has a long history of providing evidence for its positive effects on the course of multiple cancers. However, in spite of the poor prognosis and unacceptable 5-year survival rates, the actions mediated by melatonin in oral tumors have been scarcely investigated. Nevertheless, the sporadic studies that have been carried out have reported promising oncostatic effects of melatonin in oral cancer, the potentiation of radiochemotherapy toxicity, the inhibition of drug resistance and the amelioration of patient’s wellness. Moreover, the reversion of genome methylation, chromatin modifications and expressed miRNAs until their physiological demarcations highlighted by recent epigenomics, has accentuated the utility of adjuvant melatonin. Additionally, the functional cancellation by hypermethylation of melatonin receptor 1A gene suggests melatonin signaling as tumor suppressive in oral cancer. Therefore, systematic clinical programmes that help to discern the therapeutic potential of melatonin in oral cancer are needed. 

## Figures and Tables

**Table 1:** Melatonin administration as an adjuvant for the management of oral cancer *in vitro*, *in vivo* and in human studies.

	Model	Melatonin Combined with Other Drugs	Effect	Reference
	Cal 27 and SCC-9 human squamous cell carcinoma lines	MLT (0.1, 0.5 or 1 mM) plus rapamycin (20 nM)	Enhanced cytotoxic effects of rapamycin in HNSCC cells	[16]
*In vitro* studies	Cal 27 and SCC-9 human squamous cell carcinoma lines	MLT (0.1, 0.5, 1 and 5 mM) combined with 8 Gy irradiation and 10 µM Cisplatin	Improved effectiveness of chemo- and radiotherapy	[17]
	OSCC cells	MLT (0,5 and 5 mM) combined with 5-FU (1 and 10 µM)	Potentiated cytotoxicity of 5-FU	[57]
	Male Wistar rats	7.5 Gy to oral mucosa for 5 days plus 3% MLT gel for 21 days post-irradiation	Inhibited radiotherapy-induced mucositis	[53]
*In vivo* studies	OSCC-xenografted mice	MLT (20 mg/kg/day) combined with 5-FU (20 mg/kg, twice per week) for 4 weeks	Inhibited OSCC tumor growth	[57]
	Cal 27 cells -xenografted rats	Rapamycin (1 mg/kg i.p.) for 10 days plus injection of MLT (300 mg/kg s.c.) 1 day before each rapamycin administration	Ameliorated the toxicity of rapamycin in normal cells	[16]
	Clinical trial in 27 patients with metastatic solid tumor in HNC	MLT (20 mg/day orally every day) combined with 5-FU and Cisplatin	Increased 1-year survival and tumor regression rate; and side effects reduced	[56]
Human studies	Clinical trial in 39 patients with HNC under concurrent chemoradiation	MLT gargle (20 mg) before irradiation, and MLT capsules (20 mg) taken before bedtime during 7 weeks of concurrent chemoradiation	Delayed onset of oral mucositis and reduced amount of morphine required to alleviate the pain vs controls	[58]

MLT: Melatonin, i.p.: Intraperitoneally, s.c.: Subcutaneously, Gy: Gray, SCC-9: Squamous cell carcinoma-9, OSCC: Oral squamous cell carcinoma, HNSCC: Head and neck squamous cell carcinoma, HNC: Head and neck cancers, 5-FU: 5-fluorouracil.

**Table 2:** Adjuvant use of melatonin in ameliorating radio/chemotherapy side-effects.

Oncologic Treatment	Pathological Complication	Melatonin Treatment	Clinical Observations	References
Concurrent chemoradiation (5 days/week of radiation for 7 weeks; total dose ≥50 Gy), plus cisplatin chemotherapy	Oral mucositis	Oral gargle (20 mg/10 mL or 20 mg oral dose)	Adjuvant MLT delayed the onset of oral mucositis, reducing the palliative morphine required to control pain.	[58]
Male Wistar rats irradiated under anesthesia with a dose of 7.5 Gy/day for 5 days	Oral mucositis	45 mg/day for 21 days postirradiation, either by local mouth application (MLT gel; 48 h before each irradiation, 3 times/day) or by s.c. injection each day	MLT prevented mucosal disruption and ulcer formation by blunting inflammasome signaling activation in the tongue	[53]

MLT: Melatonin, s.c.: Subcutaneously, Gy: Gray.

**Table 3:** Studies supporting the use of melatonin on epigenetic modulation of OC.

Epigenetic Control	Experimental Model	Melatonin Treatment	Main Findings	References
**DNA methylation**	OSCC cell lines		The loss by homozygous deletion or silencing by CpG hypermethylation of the MLT receptor 1A (MTNR1A) gene was associated with cancer status and tumor phenotype	[50]
**Histone modification**	Patient-derived tumor xenografts models overexpressing LSD1. Mouse-based subcutaneous OC SCC25-xenograft model. OSCC cell lines.	20 mg/kg daily, i.p., for 24 and 42 days. 0–20 mM for 24 h 2–4 mM for 24 and 48 h	MLT demonstrated anti-OC activity through LSD1 down-regulation	[87]
	HSC-3 and OECM-1 OC cell lines	1 mM for 24 h	MLT inhibited migration of tumor cells through down-regulation of MMP-9 expression and activity by decreasing CREBBP/EP300-dependent H3 and H4 histone acetylation on MMP-9 promoter	[90]
**Promoter activity**	HONE-1, NPC-39 and NPC-BM nasopharyngeal carcinoma cell lines.	0.5–1 mM	MLT reduced MMP-9 promoter activity through inhibition of SP-1 transcription factor expression	[91]
	SCC9, SCC25 and CAL27 OSCC cell lines.	10 μg/mL for 72 h	MLT reduced miR-155 and increased miR-21.	[102]
**Non-coding micro-RNAs**	121 OC specimens and 66 normal counterparts for the study of miR-24 expression HCT 116 and MCF-7 cells.	1 μM for 72 h	MLT decreased miR-24 expression, which pairs with the regulation of cell proliferation, DNA damage and oncogenic transformation genes.	[100]

MLT: Melatonin, OC: Oral cancer, OSCC: Oral squamous-cell carcinoma, s.c.: Subcutaneously, i.p.: Intraperitoneally, MMP-9: Metalloproteinase 9, LSD1: Histone lysine-specific demethylase, SP-1: Specificity protein-1, CREBBP/EP300: CREB binding protein/ E1A binding protein P300.

## References

[1] Warnakulasuriya S. (2009). Global epidemiology of oral and oropharyngeal cancer. Oral Oncol..

[2] Chin D., Boyle G.M., Porceddu S., Theile D.R., Parsons P.G., Coman W.B. (2006). Head and neck cancer: Past, present and future. Expert Rev. Anticancer Ther..

[3] Montero P.H., Patel S.G. (2015). Cancer of the oral cavity. Surg. Oncol. Clin. N. Am..

[4] Van der Waal I., de Bree R., Brakenhoff R., Coebergh J.W. (2011). Early diagnosis in primary oral cancer: Is it possible?. Med. Oral Patol. Oral Cir. Bucal.

[5] Cancer Genome Atlas N. (2015). Comprehensive genomic characterization of head and neck squamous cell carcinomas. Nature.

[6] Razzouk S. (2014). Translational genomics and head and neck cancer: Toward precision medicine. Clin. Genet..

[7] Viet C.T., Schmidt B.L. (2010). Understanding oral cancer in the genome era. Head Neck.

[8] Li C.C., Shen Z., Bavarian R., Yang F., Bhattacharya A. (2018). Oral cancer: Genetics and the role of precision medicine. Dent. Clin. N. Am..

[9] Lo W.L., Kao S.Y., Chi L.Y., Wong Y.K., Chang R.C. (2003). Outcomes of oral squamous cell carcinoma in Taiwan after surgical therapy: Factors affecting survival. J. Oral Maxillofac. Surg..

[10] Zhang X., Li L., Wei D., Yap Y., Chen F. (2007). Moving cancer diagnostics from bench to bedside. Trends Biotechnol..

[11] Cutando A., Gomez-Moreno G., Arana C., Acuna-Castroviejo D., Reiter R.J. (2007). Melatonin: Potential functions in the oral cavity. J. Periodontol..

[12] Laakso M.L., Porkka-Heiskanen T., Alila A., Stenberg D., Johansson G. (1990). Correlation between salivary and serum melatonin: Dependence on serum melatonin levels. J. Pineal Res..

[13] Praninskiene R., Dumalakiene I., Kemezys R., Mauricas M., Jucaite A. (2012). Diurnal melatonin patterns in children: Ready to apply in clinical practice?. Pediatr. Neurol..

[14] Russo D., Merolla F., Varricchio S., Salzano G., Zarrilli G., Mascolo M., Strazzullo V., Di Crescenzo R.M., Celetti A., Ilardi G. (2018). Epigenetics of oral and oropharyngeal cancers. Biomed. Rep..

[15] Kulkarni V., Uttamani J.R., Naqvi A.R., Nares S. (2017). microRNAs: Emerging players in oral cancers and inflammatory disorders. Tumour Biol..

[16] Shen Y.Q., Guerra-Librero A., Fernández-Gil B.I., Florido J., Garcia-Lopez S., Martínez-Ruiz L., Mendivil-Perez M., Soto-Mercado V., Acuna-Castroviejo D., Ortega-Arellano H. (2018). Combination of melatonin and rapamycin for head and neck cancer therapy: Suppression of AKT/mTOR pathway activation, and activation of mitophagy and apoptosis via mitochondrial function regulation. J. Pineal Res..

[17] Fernández-Gil B.I., Guerra-Librero A., Shen Y.Q., Florido J., Martínez-Ruiz L., García-López S., Adan C., Rodríguez-Santana C., Acuna-Castroviejo D., Quinones-Hinojosa A. (2019). Melatonin enhances cisplatin and radiation cytotoxicity in head and neck squamous cell carcinoma by stimulating mitochondrial ROS generation, apoptosis, and autophagy. Oxid. Med. Cell Longev..

[18] Bondy S.C., Campbell A. (2018). Mechanisms underlying tumor suppressive properties of melatonin. Int. J. Mol. Sci..

[19] Reiter R.J., Rosales-Corral S.A., Liu X.Y., Acuna-Castroviejo D., Escames G., Tan D.X. (2015). Melatonin in the oral cavity: Physiological and pathological implications. J. Periodontal Res..

[20] Leoncini E., Vukovic V., Cadoni G., Pastorino R., Arzani D., Bosetti C., Canova C., Garavello W., La Vecchia C., Maule M. (2015). Clinical features and prognostic factors in patients with head and neck cancer: Results from a multicentric study. Cancer Epidemiol..

[21] Gatta G., Botta L., Sanchez M.J., Anderson L.A., Pierannunzio D., Licitra L., Group E.W. (2015). Prognoses and improvement for head and neck cancers diagnosed in Europe in early 2000s: The EUROCARE-5 population-based study. Eur. J. Cancer.

[22] Siegel R., Ma J., Zou Z., Jemal A. (2014). Cancer statistics, 2014. CA Cancer J. Clin..

[23] Siegel R.L., Miller K.D., Jemal A. (2019). Cancer statistics, 2019. CA Cancer J. Clin..

[24] D’Souza S., Addepalli V. (2018). Preventive measures in oral cancer: An overview. Biomed. Pharmacother..

[25] Lambert R., Sauvaget C., de Camargo Cancela M., Sankaranarayanan R. (2011). Epidemiology of cancer from the oral cavity and oropharynx. Eur. J. Gastroenterol. Hepatol..

[26] Chaturvedi A.K., Engels E.A., Pfeiffer R.M., Hernandez B.Y., Xiao W., Kim E., Jiang B., Goodman M.T., Sibug-Saber M., Cozen W. (2011). Human papillomavirus and rising oropharyngeal cancer incidence in the United States. J. Clin. Oncol..

[27] D’Souza G., Anantharaman D., Gheit T., Abedi-Ardekani B., Beachler D.C., Conway D.I., Olshan A.F., Wunsch-Filho V., Toporcov T.N., Ahrens W. (2016). Effect of HPV on head and neck cancer patient survival, by region and tumor site: A comparison of 1362 cases across three continents. Oral Oncol..

[28] Lydiatt W.M., Patel S.G., O’Sullivan B., Brandwein M.S., Ridge J.A., Migliacci J.C., Loomis A.M., Shah J.P. (2017). Head and Neck cancers-major changes in the American Joint Committee on cancer eighth edition cancer staging manual. CA Cancer J. Clin..

[29] Monteiro L.S., Amaral J.B., Vizcaino J.R., Lopes C.A., Torres F.O. (2014). A clinical-pathological and survival study of oral squamous cell carcinomas from a population of the North of Portugal. Med. Oral Patol. Oral Cir. Bucal.

[30] Weijers M., Leemans C.R., Aartman I.H., Karagozoglu K.H., van der Waal I. (2011). Oral cancer trends in a single head-and-neck cancer center in the Netherlands; decline in T-stage at the time of admission. Med. Oral Patol. Oral Cir. Bucal.

[31] Tiwana M.S., Wu J., Hay J., Wong F., Cheung W., Olson R.A. (2014). 25 year survival outcomes for squamous cell carcinomas of the head and neck: Population-based outcomes from a Canadian province. Oral Oncol..

[32] Chang J.H., Wu C.C., Yuan K.S., Wu A.T.H., Wu S.Y. (2017). Locoregionally recurrent head and neck squamous cell carcinoma: Incidence, survival, prognostic factors, and treatment outcomes. Oncotarget.

[33] Dias F.L., Kligerman J., Matos de Sa G., Arcuri R.A., Freitas E.Q., Farias T., Matos F., Lima R.A. (2001). Elective neck dissection versus observation in stage I squamous cell carcinomas of the tongue and floor of the mouth. Otolaryngol. Head Neck Surg..

[34] Ferlito A., Rinaldo A., Robbins K.T., Leemans C.R., Shah J.P., Shaha A.R., Andersen P.E., Kowalski L.P., Pellitteri P.K., Clayman G.L. (2003). Changing concepts in the surgical management of the cervical node metastasis. Oral Oncol..

[35] D’Cruz A.K., Vaish R., Dhar H. (2018). Oral cancers: Current status. Oral Oncol..

[36] Li Y., Li S., Zhou Y., Meng X., Zhang J.J., Xu D.P., Li H.B. (2017). Melatonin for the prevention and treatment of cancer. Oncotarget.

[37] Gil-Martin E., Egea J., Reiter R.J., Romero A. (2019). The emergence of melatonin in oncology: Focus on colorectal cancer. Med. Res. Rev..

[38] Zare H., Shafabakhsh R., Reiter R.J., Asemi Z. (2019). Melatonin is a potential inhibitor of ovarian cancer: Molecular aspects. J. Ovarian Res..

[39] Liu R., Wang H.L., Deng M.J., Wen X.J., Mo Y.Y., Chen F.M., Zou C.L., Duan W.F., Li L., Nie X. (2018). Melatonin inhibits reactive oxygen species-driven proliferation, epithelial-mesenchymal transition, and vasculogenic mimicry in oral cancer. Oxid. Med. Cell Longev..

[40] Gonçalves Ndo N., Rodrigues R.V., Jardim-Perassi B.V., Moschetta M.G., Lopes J.R., Colombo J., Zuccari D.A. (2014). Molecular markers of angiogenesis and metastasis in lines of oral carcinoma after treatment with melatonin. Anticancer Agents Med. Chem..

[41] Cutando A., López-Valverde A., De Vicente J., Gimenez J.L., CarcÍa I.A., De Diego R.G. (2014). Action of melatonin on squamous cell carcinoma and other tumors of the oral cavity (Review). Oncol. Lett..

[42] Lv J.W., Zheng Z.Q., Wang Z.X., Zhou G.Q., Chen L., Mao Y.P., Lin A.H., Reiter R.J., Ma J., Chen Y.P. (2019). Pan-cancer genomic analyses reveal prognostic and immunogenic features of the tumor melatonergic microenvironment across 14 solid cancer types. J. Pineal Res..

[43] Mehta A., Kaur G. (2014). Potential role of melatonin in prevention and treatment of oral carcinoma. Indian J. Dent..

[44] Jockers R., Delagrange P., Dubocovich M.L., Markus R.P., Renault N., Tosini G., Cecon E., Zlotos D.P. (2016). Update on melatonin receptors: IUPHAR Review 20. Br. J. Pharmacol..

[45] Slominski R.M., Reiter R.J., Schlabritz-Loutsevitch N., Ostrom R.S., Slominski A.T. (2012). Melatonin membrane receptors in peripheral tissues: Distribution and functions. Mol. Cell. Endocrinol..

[46] Isola M., Lilliu M.A. (2016). Melatonin localization in human salivary glands. J. Oral Pathol. Med..

[47] Cengiz M.I., Cengiz S., Wang H.L. (2012). Melatonin and oral cavity. Int. J. Dent..

[48] Shimozuma M., Tokuyama R., Tatehara S., Umeki H., Ide S., Mishima K., Saito I., Satomura K. (2011). Expression and cellular localizaion of melatonin-synthesizing enzymes in rat and human salivary glands. Histochem. Cell Biol..

[49] Lin F.Y., Lin C.W., Yang S.F., Lee W.J., Lin Y.W., Lee L.M., Chang J.L., Weng W.C., Lin C.H., Chien M.H. (2015). Interactions between environmental factors and melatonin receptor type 1A polymorphism in relation to oral cancer susceptibility and clinicopathologic development. PLoS ONE.

[50] Nakamura E., Kozaki K., Tsuda H., Suzuki E., Pimkhaokham A., Yamamoto G., Irie T., Tachikawa T., Amagasa T., Inazawa J. (2008). Frequent silencing of a putative tumor suppressor gene melatonin receptor 1 A (MTNR1A) in oral squamous-cell carcinoma. Cancer Sci..

[51] Cutando A., Aneiros-Fernandez J., Lopez-Valverde A., Arias-Santiago S., Aneiros-Cachaza J., Reiter R.J. (2011). A new perspective in oral health: Potential importance and actions of melatonin receptors MT1, MT2, MT3, and RZR/ROR in the oral cavity. Arch. Oral Biol..

[52] Fan T., Pi H., Li M., Ren Z., He Z., Zhu F., Tian L., Tu M., Xie J., Liu M. (2018). Inhibiting MT2-TFE3-dependent autophagy enhances melatonin-induced apoptosis in tongue squamous cell carcinoma. J. Pineal Res..

[53] Ortiz F., Acuna-Castroviejo D., Doerrier C., Dayoub J.C., López L.C., Venegas C., García J.A., López A., Volt H., Luna-Sánchez M. (2015). Melatonin blunts the mitochondrial/NLRP3 connection and protects against radiation-induced oral mucositis. J. Pineal Res..

[54] Foley H.M., Steel A.E. (2019). Adverse events associated with oral administration of melatonin: A critical systematic review of clinical evidence. Complement. Ther. Med..

[55] William W.N. (2012). Oral premalignant lesions: Any progress with systemic therapies?. Curr. Opin. Oncol..

[56] Lissoni P., Barni S., Mandala M., Ardizzoia A., Paolorossi F., Vaghi M., Longarini R., Malugani F., Tancini G. (1999). Decreased toxicity and increased efficacy of cancer chemotherapy using the pineal hormone melatonin in metastatic solid tumour patients with poor clinical status. Eur. J. Cancer.

[57] Lu Y.X., Chen D.L., Wang D.S., Chen L.Z., Mo H.Y., Sheng H., Bai L., Wu Q.N., Yu H.E., Xie D. (2016). Melatonin enhances sensitivity to fluorouracil in oesophageal squamous cell carcinoma through inhibition of Erk and Akt pathway. Cell Death Dis..

[58] Onseng K., Johns N.P., Khuayjarernpanishk T., Subongkot S., Priprem A., Hurst C., Johns J. (2017). Beneficial effects of adjuvant melatonin in minimizing oral mucositis complications in head and neck cancer patients receiving concurrent chemoradiation. J. Altern. Complement. Med..

[59] Ha P.K., Califano J.A. (2006). Promoter methylation and inactivation of tumour-suppressor genes in oral squamous-cell carcinoma. Lancet Oncol..

[60] Potaczek D.P., Harb H., Michel S., Alhamwe B.A., Renz H., Tost J. (2017). Epigenetics and allergy: From basic mechanisms to clinical applications. Epigenetics.

[61] Lee J.K., Kim M.J., Hong S.P., Hong S.D. (2004). Inactivation patterns of p16/INK4A in oral squamous cell carcinomas. Exp. Mol. Med..

[62] Saito K., Uzawa K., Endo Y., Kato Y., Nakashima D., Ogawara K., Shiba M., Bukawa H., Yokoe H., Tanzawa H. (2006). Plasma membrane Ca2+ ATPase isoform 1 down-regulated in human oral cancer. Oncol. Rep..

[63] Shaw R. (2006). The epigenetics of oral cancer. Int. J. Oral Maxillofac. Surg..

[64] Smith I.M., Mydlarz W.K., Mithani S.K., Califano J.A. (2007). DNA global hypomethylation in squamous cell head and neck cancer associated with smoking, alcohol consumption and stage. Int. J. Cancer.

[65] Ghantous Y., Schussel J.L., Brait M. (2018). Tobacco and alcohol-induced epigenetic changes in oral carcinoma. Curr. Opin. Oncol..

[66] Shaw R.J., Liloglou T., Rogers S.N., Brown J.S., Vaughan E.D., Lowe D., Field J.K., Risk J.M. (2006). Promoter methylation of P16, RARbeta, E-cadherin, cyclin A1 and cytoglobin in oral cancer: Quantitative evaluation using pyrosequencing. Br. J. Cancer.

[67] Shaw R.J., Hall G.L., Lowe D., Bowers N.L., Liloglou T., Field J.K., Woolgar J.A., Risk J.M. (2007). CpG island methylation phenotype (CIMP) in oral cancer: Associated with a marked inflammatory response and less aggressive tumour biology. Oral Oncol..

[68] Zhong L., Liu Y., Wang K., He Z., Gong Z., Zhao Z., Yang Y., Gao X., Li F., Wu H. (2018). Biomarkers: Paving stones on the road towards the personalized precision medicine for oral squamous cell carcinoma. BMC Cancer.

[69] Morandi L., Gissi D., Tarsitano A., Asioli S., Gabusi A., Marchetti C., Montebugnoli L., Foschini M.P. (2017). CpG location and methylation level are crucial factors for the early detection of oral squamous cell carcinoma in brushing samples using bisulfite sequencing of a 13-gene panel. Clin. Epigenet..

[70] Bai G., Song J., Yuan Y., Chen Z., Tian Y., Yin X., Niu Y., Liu J. (2019). Systematic analysis of differentially methylated expressed genes and site-speci fi c methylation as potential prognostic markers in head and neck cancer. J. Cell Physiol..

[71] Zhou C., Ye M., Ni S., Li Q., Ye D., Li J., Shen Z., Deng H. (2018). DNA methylation biomarkers for head and neck squamous cell carcinoma. Epigenetics.

[72] Arayataweegool A., Srisuttee R., Mahattanasakul P., Tangjaturonsasme N., Kerekhanjanarong V., Kitkumthorn N., Mutirangura A. (2019). Head and neck squamous cell carcinoma drives long interspersed element-1 hypomethylation in the peripheral blood mononuclear cells. Oral Dis..

[73] Haim A., Zubidat A.E. (2015). Artificial light at night: Melatonin as a mediator between the environment and epigenome. Philos. Trans. R. Soc. Lond. B Biol. Sci..

[74] Cajochen C., Jud C., Munch M., Kobialka S., Wirz-Justice A., Albrecht U. (2006). Evening exposure to blue light stimulates the expression of the clock gene PER2 in humans. Eur. J. Neurosci..

[75] Rana S., Mahmood S. (2010). Circadian rhythm and its role in malignancy. J. Circadian Rhythm..

[76] Hardeland R. (2017). Melatonin and the pathologies of weakened or dysregulated circadian oscillators. J. Pineal Res..

[77] Schwimmer H., Metzer A., Pilosof Y., Szyf M., Machnes Z.M., Fares F., Harel O., Haim A. (2014). Light at night and melatonin have opposite effects on breast cancer tumors in mice assessed by growth rates and global DNA methylation. Chronobiol. Int..

[78] Zubidat A.E., Fares B., Fares F., Haim A. (2018). Artificial light at night of different spectral compositions differentially affects tumor growth in mice: Interaction with melatonin and epigenetic pathways. Cancer Control..

[79] Agbaria S., Haim A., Fares F., Zubidat A.E. (2019). Epigenetic modification in 4T1 mouse breast cancer model by artificial light at night and melatonin—the role of DNA-methyltransferase. Chronobiol. Int..

[80] Smiraglia D.J., Smith L.T., Lang J.C., Rush L.J., Dai Z., Schuller D.E., Plass C. (2003). Differential targets of CpG island hypermethylation in primary and metastatic head and neck squamous cell carcinoma (HNSCC). J. Med. Genet..

[81] Von Zeidler S.V., Miracca E.C., Nagai M.A., Birman E.G. (2004). Hypermethylation of the p16 gene in normal oral mucosa of smokers. Int. J. Mol. Med..

[82] Kresty L.A., Mallery S.R., Knobloch T.J., Song H., Lloyd M., Casto B.C., Weghorst C.M. (2002). Alterations of p16(INK4a) and p14(ARF) in patients with severe oral epithelial dysplasia. Cancer Res..

[83] Kulkarni V., Saranath D. (2004). Concurrent hypermethylation of multiple regulatory genes in chewing tobacco associated oral squamous cell carcinomas and adjacent normal tissues. Oral Oncol..

[84] Eberharter A., Becker P.B. (2002). Histone acetylation: A switch between repressive and permissive chromatin. Second in review series on chromatin dynamics. EMBO Rep..

[85] Webber L.P., Wagner V.P., Curra M., Vargas P.A., Meurer L., Carrard V.C., Squarize C.H., Castilho R.M., Martins M.D. (2017). Hypoacetylation of acetyl-histone H3 (H3K9ac) as marker of poor prognosis in oral cancer. Histopathology.

[86] Yuan C., Li Z., Qi B., Zhang W., Cheng J., Wang Y. (2015). High expression of the histone demethylase LSD1 associates with cancer cell proliferation and unfavorable prognosis in tongue cancer. J. Oral Pathol. Med..

[87] Yang C.Y., Lin C.K., Tsao C.H., Hsieh C.C., Lin G.J., Ma K.H., Shieh Y.S., Sytwu H.K., Chen Y.W. (2017). Melatonin exerts anti-oral cancer effect via suppressing LSD1 in patient-derived tumor xenograft models. Oncotarget.

[88] Chung Y.L., Lee M.Y., Pui N.N. (2009). Epigenetic therapy using the histone deacetylase inhibitor for increasing therapeutic gain in oral cancer: Prevention of radiation-induced oral mucositis and inhibition of chemical-induced oral carcinogenesis. Carcinogenesis.

[89] Abdel Moneim A.E., Guerra-Librero A., Florido J., Shen Y.Q., Fernández-Gil B., Acuna-Castroviejo D., Escames G. (2017). Oral mucositis: Melatonin gel an effective new treatment. Int. J. Mol. Sci..

[90] Yeh C.M., Lin C.W., Yang J.S., Yang W.E., Su S.C., Yang S.F. (2016). Melatonin inhibits TPA-induced oral cancer cell migration by suppressing matrix metalloproteinase-9 activation through the histone acetylation. Oncotarget.

[91] Ho H.Y., Lin C.W., Chien M.H., Reiter R.J., Su S.C., Hsieh Y.H., Yang S.F. (2016). Melatonin suppresses TPA-induced metastasis by downregulating matrix metalloproteinase-9 expression through JNK/SP-1 signaling in nasopharyngeal carcinoma. J. Pineal Res..

[92] Selbach M., Schwanhausser B., Thierfelder N., Fang Z., Khanin R., Rajewsky N. (2008). Widespread changes in protein synthesis induced by microRNAs. Nature.

[93] Sethi N., Wright A., Wood H., Rabbitts P. (2014). MicroRNAs and head and neck cancer: Reviewing the first decade of research. Eur. J. Cancer.

[94] Kolokythas A., Miloro M., Zhou X. (2011). Review of microRNA proposed target genes in oral cancer. Part II. J. Oral Maxillofac. Res..

[95] Manasa V.G., Kannan S. (2017). Impact of microRNA dynamics on cancer hallmarks: An oral cancer scenario. Tumour Biol..

[96] Schneider A., Victoria B., Lopez Y.N., Suchorska W., Barczak W., Sobecka A., Golusinski W., Masternak M.M., Golusinski P. (2018). Tissue and serum microRNA profile of oral squamous cell carcinoma patients. Sci. Rep..

[97] Min A., Zhu C., Peng S., Rajthala S., Costea D.E., Sapkota D. (2015). MicroRNAs as important players and biomarkers in oral carcinogenesis. Biomed. Res. Int..

[98] Lin S.C., Liu C.J., Lin J.A., Chiang W.F., Hung P.S., Chang K.W. (2010). miR-24 up-regulation in oral carcinoma: Positive association from clinical and in vitro analysis. Oral Oncol..

[99] Lee S.E., Kim S.J., Youn J.P., Hwang S.Y., Park C.S., Park Y.S. (2011). MicroRNA and gene expression analysis of melatonin-exposed human breast cancer cell lines indicating involvement of the anticancer effect. J. Pineal Res..

[100] Mori F., Ferraiuolo M., Santoro R., Sacconi A., Goeman F., Pallocca M., Pulito C., Korita E., Fanciulli M., Muti P. (2016). Multitargeting activity of miR-24 inhibits long-term melatonin anticancer effects. Oncotarget.

[101] Lee S.E., Kim S.J., Yoon H.J., Yu S.Y., Yang H., Jeong S.I., Hwang S.Y., Park C.S., Park Y.S. (2013). Genome-wide profiling in melatonin-exposed human breast cancer cell lines identifies differentially methylated genes involved in the anticancer effect of melatonin. J. Pineal Res..

[102] Hunsaker M., Barba G., Kingsley K., Howard K.M. (2019). Differential microRNA expression of miR-21 and miR-155 within oral cancer extracellular vesicles in response to melatonin. Dent. J..

[103] Avissar M., Christensen B.C., Kelsey K.T., Marsit C.J. (2009). MicroRNA expression ratio is predictive of head and neck squamous cell carcinoma. Clin. Cancer Res..

[104] Lamperska K.M., Kozlowski P., Kolenda T., Teresiak A., Blizniak R., Przybyla W., Masternak M.M., Golusinski P., Golusinski W. (2016). Unpredictable changes of selected miRNA in expression profile of HNSCC. Cancer Biomark..

[105] Cervigne N.K., Reis P.P., Machado J., Sadikovic B., Bradley G., Galloni N.N., Pintilie M., Jurisica I., Perez-Ordonez B., Gilbert R. (2009). Identification of a microRNA signature associated with progression of leukoplakia to oral carcinoma. Hum. Mol. Genet..

[106] He Q., Chen Z., Cabay R.J., Zhang L., Luan X., Chen D., Yu T., Wang A., Zhou X. (2016). microRNA-21 and microRNA-375 from oral cytology as biomarkers for oral tongue cancer detection. Oral Oncol..

[107] Zeljic K., Jovanovic I., Jovanovic J., Magic Z., Stankovic A., Supic G. (2018). MicroRNA meta-signature of oral cancer: Evidence from a meta-analysis. Upsala J. Med. Sci..

[108] Yan Z.Y., Luo Z.Q., Zhang L.J., Li J., Liu J.Q. (2017). Integrated analysis and microRNA expression profiling identified seven miRNAs associated with progression of oral squamous cell carcinoma. J. Cell. Physiol..

[109] Gissi D.B., Morandi L., Gabusi A., Tarsitano A., Marchetti C., Cura F., Palmieri A., Montebugnoli L., Asioli S., Foschini M.P. (2018). A noninvasive test for microRNA expression in oral squamous cell carcinoma. Int. J. Mol. Sci..

[110] Shah S., Jadhav K., Shah V., Gupta N., Dagrus K. (2016). miRNA 21: Diagnostic prognostic and therapeutic marker for oral cancer. Microrna.

[111] Yap T., Koo K., Cheng L., Vella L.J., Hill A.F., Reynolds E., Nastri A., Cirillo N., Seers C., McCullough M. (2018). Predicting the presence of oral squamous cell carcinoma using commonly dysregulated MicroRNA in oral swirls. Cancer Prev. Res..

